# Effectiveness of anti-TNF-α agents in the treatment of refractory juvenile dermatomyositis

**DOI:** 10.1186/1546-0096-9-S1-O29

**Published:** 2011-09-14

**Authors:** EL Boulter, L Beard, C Ryder, CA Pilkington

**Affiliations:** 1 Rheumatology Department, Great Ormond Street Hospital, London, UK; 2 Rheumatology Unit, UCL Institute of Child Health, University College London, UK; 3 Rheumatology Department, Birmingham Children’s Hospital NHS Foundation Trust, UK

## Background

Juvenile dermatomyositis (JDM) is a rare, chronic inflammatory disease. Anti-TNF-α agents are increasingly being used to treat disease that is refractory to other treatments. There is a lack of literature regarding the effectiveness of anti-TNF-α agents in JDM.

## Aim

To assess the response of refractory JDM patients to anti-TNF-α agents.

## Methods

The Juvenile Dermatomyositis National (UK and Ireland) Cohort Biomarker Study and Repository for Idiopathic Inflammatory Myopathies database was searched for patients treated with anti-TNF-α agents.

## Results

INFLIXIMAB: 28/30(93%) patients had data available. Indicators for starting infliximab were: muscle weakness (70%), non-ulcerative skin disease (57%), calcinosis (33%), and nail fold changes (30%). All patients with a low CMAS (n=18) improved. Physician VAS improved in 20/26(77%), CHAQ score in 11/16(69%), skin disease in 19/23(83%), calcinosis in 6/13(46%) and muscle enzymes in 6/10(60%). Prednisolone dose decreased in 17/21(81%).

ADALIMUMAB: 10/11(91%) had previously been treated with infliximab. 6/10(60%) were changed to improve disease control: 4 had persistent skin disease, 2 improved; 3 had progressive calcinosis, 1 improved, 2 remained stable. 8/9(89%) maintained previous gains or made further improvement in other parameters other than skin and calcinosis.

ETANERCEPT: 4/7(57%) patients had data available. 2 improved, 2 were switched to infliximab, one for increasing calcinosis, one for compliance issues.

## Conclusions

Infliximab provides clinical benefit to patients with JDM refractory to other treatments; particularly muscle weakness. Switching to adalimumab benefited some patients; gains made on infliximab were maintained in most cases.

